# Costs of endovascular and open repair of thoracic aortic aneurysms

**DOI:** 10.1093/bjs/znad378

**Published:** 2023-12-13

**Authors:** Joanne Gray, Andrew McCarthy, Dilupa Samarakoon, Peter McMeekin, Linda Sharples, Priya Sastry, Paul Crawshaw, Colin Bicknell, the ETTAA Collaborative Group, Stephen Large, Linda Sharples, Luke Vale, Priya Sastry, Colin Bicknell, Carol Freeman, Andrew Cook, Yi-Da Chiu, Andrew McCarthy, Jo Gray, Peter McMeekin, S Rao Vallabhaneni, Nicky Watson, Dilupa Samarakoon, Thomas Devine, Tom Duffy, Victoria Hughes

**Affiliations:** Faculty of Health and Life Sciences, Northumbria University, Newcastle Upon Tyne, UK; Faculty of Health and Life Sciences, Northumbria University, Newcastle Upon Tyne, UK; Faculty of Health and Life Sciences, Northumbria University, Newcastle Upon Tyne, UK; Faculty of Health and Life Sciences, Northumbria University, Newcastle Upon Tyne, UK; Department of Medical Statistics, London School of Hygiene and Tropical Medicine, London, UK; Department of Cardiac Surgery, John Radcliffe Hospital, Oxford University Hospitals, Oxford, UK; School of Social Sciences, Humanities and Law, Teesside University, Middlesbrough, UK; Department of Surgery and Cancer, Imperial College, London, UK; Imperial Vascular Unit, Imperial Healthcare NHS Trust, London, UK

## Abstract

**Background:**

Repair of thoracic aortic aneurysms with either endovascular repair (TEVAR) or open surgical repair (OSR) represents major surgery, is costly and associated with significant complications. The aim of this study was to establish accurate costs of delivering TEVAR and OSR in a cohort of UK NHS patients suitable for open and endovascular treatment for the whole treatment pathway from admission and to discharge and 12-month follow-up.

**Methods:**

A prospective study of UK NHS patients from 30 NHS vascular/cardiothoracic units in England aged ≥18, with distal arch/descending thoracic aortic aneurysms (CTAA) was undertaken. A multicentre prospective cost analysis of patients (recruited March 2014–July 2018, follow-up until July 2019) undergoing TEVAR or OSR was performed. Patients deemed suitable for open or endovascular repair were included in this study. A micro-costing approach was adopted.

**Results:**

Some 115 patients having undergone TEVAR and 35 patients with OSR were identified. The mean (s.d.) cost of a TEVAR procedure was higher £26 536 (£9877) *versus* OSR £17 239 (£8043). Postoperative costs until discharge were lower for TEVAR £7484 (£7848) *versus* OSR £28 636 (£23 083). Therefore, total NHS costs from admission to discharge were lower for TEVAR £34 020 (£14 301), *versus* OSR £45 875 (£43 023). However, mean NHS costs for 12 months following the procedure were slightly higher for the TEVAR £5206 (£11 585) *versus* OSR £5039 (£11 994).

**Conclusions:**

Surgical procedure costs were higher for TEVAR due to device costs. Total in-hospital costs were higher for OSR due to longer hospital and critical care stay. Follow-up costs over 12 months were slightly higher for TEVAR due to hospital readmissions.

## Teaser

Detailed micro-costing of endovascular stent grafting (TEVAR) and open surgical repair (OSR) in a cohort of UK NHS patients for whole of treatment pathway up to 12 months follow-up. Overall costs are higher for OSR, driven by length of stay and critical care post-procedure, compared to TEVAR, with costs driven by stent graft devices. These detailed micro-costing estimates exceed current NHS tariffs for reimbursement.

## Introduction

Patients with chronic thoracic aortic aneurysm disease (CTAA) are most often elderly with significant and multiple co-morbidity^1,2^. Nevertheless, patients are offered major surgical repair procedures such as endovascular stent grafting (TEVAR), potentially involving expensive technology, or open surgical repair (OSR), with costly intensive care stays and rehabilitation. Despite being effective for some CTAA patients, TEVAR and OSR are associated with significant complications^3–5^. Currently, there are no UK-specific economic studies assessing outcomes beyond the chosen procedure.

There was significant controversy regarding NICE recommendations for infrarenal aneurysm repair techniques in 2019^6^. Draft recommendations, driven largely by a lack of cost effectiveness and since revised, stated that TEVAR should not be used in the elective setting. The effect of similar interventions is therefore in the spotlight. Furthermore, centralization of specialist services has been a major policy focus in the UK in recent years, with evidence that outcomes for vascular surgery can be improved through centralization^7^. Services have undergone a substantial reorganization with amalgamation of smaller units and single-handed surgeons to form larger units. However, the structured organization of complex aneurysm surgery is still in its infancy. The costs of TEVAR and OSR procedures must be accurately determined to understand the tariffs that are required to allow services to be set up and managed and to identify where resources need to be pooled into specialist complex aortic hubs. With these issues in mind, and given the increasing demand for treatment due to an ageing population with rising prevalence of CTAA^2^, and limited NHS resources, further evidence regarding accurate costs of TEVAR and OSR procedures is needed.

Cost data is an important factor in developing clinical guidelines^8,9^, yet there is a sparsity of large multicentre micro-costing studies in Europe regarding TEVAR and OSR procedures that captures the whole treatment pathway from the procedure to discharge and subsequent follow-up. The Effective Treatment of Thoracic Aortic Aneurysms (ETTAA) study provided a unique opportunity to undertake a prospective micro-costing of a large cohort of patients with CTAA repaired with open and endovascular techniques. This study aims to establish accurate costs of delivering endovascular stent TEVAR and OSR in a cohort of UK NHS patients suitable for open and endovascular treatment for whole treatment pathway from admission and to discharge and 12-month follow-up.

## Methods

### The ETTAA study

ETTAA was a large prospective observational cohort study of routine practice which recruited patients across 30 NHS vascular/cardiothoracic units in England. The protocol and funder’s report are both published^10,11^ with further details of the inclusion criteria but, briefly, consisted of patients ≥18 years of age who attended NHS hospitals in England between March 2014 and July 2018. Patients were included if they had a previously or newly diagnosed aneurysm with a diameter ≥4 cm in the arch or descending thoracic aorta. Exclusion criteria were acute dissection and previous surgical intervention for an aneurysm in the same segment of the aorta. Recruited patients were divided into four groups: watchful waits; conservative management; TEVAR; or OSR.

### The micro-costing study

Important differences between the populations undergoing TEVAR and OSR in the ETTAA study raised concerns that any comparisons are biased because of unobserved or inadequately controlled confounding^11^. Therefore, this micro-costing study was based on a subset of the larger ETTAA patient population who, based on recorded study data, had no contraindication to either OSR or TEVAR, ensuring a fair comparison in terms of patient resource use and associated costs. Eligibility to receive either procedure was assessed by clinical experts. Reasons for OSR patients being ineligible for TEVAR included: aneurysm repair extending into the ascending aorta; concomitant cardiac procedures; and unsuitable aortic morphology. Reasons for TEVAR patients being ineligible for OSR included: BMI below 20 or above 35; NYHA IV dyspnoea; or age over 85. Additionally, 10 TEVAR patients had index procedures prior to enrolment in the study; these patients were excluded to maintain equipoise.

A multicentre prospective cost analysis of ETTAA patients undergoing surgical intervention with either OSR or TEVAR for patients with CTAA was performed and reported in accordance to the Consolidated Health Economic Evaluation reporting Standards (CHEERS) statement^12^ and STROBE guidelines^13^. The micro-costing approach was adopted from the perspective of the UK NHS in order to improve the precision and accuracy of the cost estimate^14,15^ and to undertake the ‘direct enumeration and the costing of every resource input consumed in the treatment of a particular patient’^16,17^. The process involves the identification of all resources required for the provision of care; accurate measurement of each resource; and valuation of the resources used. The cost analysis focused on three stages based on chronological sequence of events: index (first) surgical procedure, post-procedure until discharge, and 12-months follow-up. For each patient, and for each stage of the cost analysis, all components of costs stratified by category of resource use were computed by multiplying units of resource use by their unit costs and summed.

Although capital equipment costs are incurred at a single point, it is typical to derive a cost for economic evaluation, whereby the initial cost of a capital asset is converted to annual equivalent sums over their expected lifetime. These annual costs are then discounted to reflect alternative investment or consumption opportunities forgone. In this study, capital equipment was discounted by 3.5 % per year^18^ and the sum of these amounts was divided by their expected annual usage to obtain a cost per procedure^19^.

### Identification and measurement of resource use

Resource utilization was identified and measured using information derived from expert clinical opinion and data collected in case report forms (CRFs), which were designed with expert guidance from members of the ETTAA collaboration including clinicians, statisticians and health economists. Details of resource use are presented in *Tables S1*, *S2*, *S3*^20^. Resources necessary to undertake the surgical procedures included staff, medical devices, reusable surgical equipment, consumables and overheads. A procedure CRF captured patient-level data on theatre time, type of graft, blood products used and perioperative complications, with other information such as surgical equipment provided by clinical experts based on a ‘typical’ procedure.

Resources necessary to provide postoperative care until discharge were collected using two CRFs. A post-procedure and discharge CRF captured the number of days in hospital, days in an intensive care unit or a high-dependency unit, postoperative blood product use, the use of any diagnostic investigations and any adverse events (including cardiac and renal failure). If a patient suffered an adverse event requiring a return to theatre, theatre time and reason for return to theatre were captured in a return-to-theatre CRF. These events were micro-costed using the same methods as described previously.

Use of NHS resources during a 12-month post-procedure follow-up including readmissions related to the aneurysm was collected at patient level using a study-specific follow-up CRF at 3, 6 and 12 months post-surgery. This included use of primary and community care and secondary care. If a patient was readmitted to hospital for reasons related to aneurysms, a hospital admission CRF captured length of stay by level of care. If a patient underwent another procedure during follow-up, this was captured using the same CRFs as the index procedure and was recorded as an additional procedure. Following discussion with experts, it was assumed that each patient who underwent TEVAR had a CT scan and a vascular outpatient visit at 1-month post discharge and annually thereafter, with OSR patients assumed to have a CT scan and a cardiology outpatient appointment at 6 months post-discharge and annually thereafter.

### Valuation of NHS resource use

#### Unit costs

Unit costs were obtained in pounds Sterling from a variety of sources including national databases^21^, published studies^22^, stent graft device manufacturers and were inflated to 2018–19 prices using the healthcare and community health services inflation index^22^. Details of unit costs are reported in *Tables S4*, *S5*, *S6*^20^.

#### Statistical analysis

Baseline characteristics were summarized with cohorts compared using Student’s *t*-test and Pearson’s χ^2^ test as appropriate. Costs for three stages (index procedure, post-procedure until discharge and follow-up) of the cost analysis were summed over all resource categories to obtain a total annual cost for each patient at 12 months. Costs were summarized and presented as mean (s.d.). Analysis was conducted using Stata v15.1.

## Results

A total of 886 patients were recruited to the ETTAA study (*Fig. S1*). Of these, 601 patients did not have any surgical procedure consisting of watchful waits (*n* = 489) and conservative management (*n* = 112). The remaining patients had at least one type of surgical procedure, TEVAR (*n* = 150) and OSR (*n* = 135), as reported elsewhere^11^. Overall, 115 TEVAR and 35 OSR patients were judged potentially eligible for both procedures (no contraindications) and were included for primary comparison. These patients were recruited from 30 ETTAA sites, with participant numbers from each site presented in *Table S8*. Of the TEVAR patients, two required aortic arch endovascular repair, 97 required descending thoracic aortic repair and 16 required complex repair with thoracoabdominal stent with fenestrated or branch grafting. Of the 35 OSR patients, 13 required hybrid grafts (for example, using frozen elephant trunk grafts), 20 patients required standard thoracic repair, and two patients required thoracoabdominal aneurysm repair. A subgroup analysis was also performed highlighting the cost differences between procedures of differing complexity.

### Patient characteristics

The patient characteristics of those deemed suitable for open and endovascular repair at baseline are presented in *Table 1*.

**Table 1:** Baseline characteristics of patients who underwent endovascular stent grafting (TEVAR) or open surgical repair (OSR) and were eligible for both procedures

Baseline characteristic	TEVAR (*n* = 115)	OSR (*n* = 35)	*P* *
Age (years)	73.1 (8.4)	62.6 (12.2)	<.001
Height (cm)	169.5 (10.0)	174.0 (10.0)	0.021
Weight (kg)	78.4 (14.5)	87.8 (18.7)	0.002
Body mass index (kg/m^2^)	26.01 (3.70)	28.10 (5.28)	0.080
Diabetes *n* (%)	11 (9.6)	0 (0)	0.068
Maximum aneurysm size (cm)	6.12 (1.20)	6.62 (1.28)	0.034
Maximum aneurysm site			0.002
Ascending aorta/arch *n* (%)	2 (1.7)	6 (17.1)	
Descending aorta/suprarenal *n* (%)	113 (98.3)	29 (82.9)	

Data are presented as mean (s.d.) unless otherwise stated.

^*^ *P* of difference between TEVAR and OSR at baseline.

### Procedure cost until discharge

Patients undergoing TEVAR (*n* = 115) had a higher estimated mean (s.d.) index procedure cost of £26 536 (£9877) compared to the estimated cost of £17 239 (£8043) for OSR patients (*n* = 35; *Table 2*). Higher costs for OSR of theatre, staff and blood products for the index procedure were outweighed by much higher costs of stent graft devices for TEVAR (TEVAR £20 966 (£9001), OSR £5461 (£6696)). Total cost for the post-procedure period until the discharge period were lower for TEVAR £7484 (£7848) compared to OSR £28 636 (£23 038). All cost categories, except for return to theatre, were higher for OSR, with much larger mean (s.d.) costs attributable to critical care (£16 391 (£20 111) *versus* £3684 (£5155)) and higher length of stays in terms of ward days (£11 113 (£22 223) *versus* £2958 (£3508)). Mean (s.d.) cost of return to theatre was slightly higher in the TEVAR group £460 (£1432) compared to the OSR group £310 (£1323). The estimated cost of the post-procedure period for OSR resulted in a higher overall cost from admission until discharge for OSR of £45 875 (£43 023) *versus* £34 020 (£14 301) for TEVAR.

**Table 2:** Costs of resource use from index procedure until discharge for patients who underwent endovascular stent grafting (TEVAR) or open surgical repair (OSR)

Costs (£)	TEVAR (*n* = 115)	OSR (*n* = 35)
**Index procedure costs**		
Stent costs*	20 966 (9001)	5461 (6696)
Theatre	3517 (1446)	6088 (1379)
Staff	1972 (1399)	3611 (1082)
Blood products	82 (349)	2079 (3378)
Total index procedure	26 536 (9877)	17 239 (8043)
**Post-procedure to discharge**		
Blood products	129 (559)	322 (625)
Diagnostic interventions	254 (335)	499 (547)
Critical care bed days†	3684 (5155)	16 391 (20 111)
Ward days	2958 (3508)	11 113 (22 223)
Return to theatre events (*n*)	17	7
Return to theatre	460 (1432)	310 (1323)
Total post-procedure to discharge	7484 (7848)	28 636 (23 083)
**Admission to discharge**		
Total NHS costs	34 020 (14 301)	45 875 (43 023)

All costs are reported in pounds Sterling (£) as mean (s.d.) unless otherwise noted.

^*^Endovascular device for TEVAR patients, surgical graft for OSR patients.

†Critical care bed days include intensive care unit and high-dependency unit.

### Subgroup analysis

Subgroup analysis was conducted to compare costs of procedures that differed in terms of complexity within TEVAR and OSR (*Table 3*). Within the TEVAR cohort, those undergoing aortic arch repair or complex repair thoracoabdominal stent with fenestrated or branch grafting had higher costs from admission until discharge £70 231 (£27 406) and £49 768 (£9 120) compared to TEVAR descending thoracic aortic repair £30 675 (£11 920). Both complex procedure types had higher cost estimated across all resource components compared to TEVAR descending thoracic aortic repair with aortic arch repair also being much higher than complex repair thoracoabdominal stent with fenestrated or branch grafting. The largest cost difference for the index procedure was due to the stent graft device cost (aortic arch £31 845 (£4320), complex repair thoracoabdominal stent with fenestrated or branch grafting £29 915 (£4753), descending thoracic aortic repair £19 266 (£8587)). The largest cost differences in the post-procedure until discharge period was due to length of stay, particularly critical care (aortic arch £16 171 (£18 860), complex repair thoracoabdominal stent with fenestrated or branch grafting £6695 (£4809), descending thoracic aortic repair £2929 (£4348)).

**Table 3:** Subgroup analysis costs of resource use from index procedure until discharge

Cost categories (£)	TEVAR (*n* = 115)			OSR (*n* = 35)		
	Aortic arch endovascular repair (*n* = 2)	Descending thoracic aortic repair (*n* = 97)	Complex repair thoracoabdominal stent with fenestrated or branch grafting (*n* = 16)	Hybrid grafts (*n* = 13)	Standard thoracic repair (*n* = 20)	Thoracoabdominal aneurysm repair (*n* = 2)
**Index procedure cost**						
Stent*	31 845 (4320)	19 266 (8587)	29 915 (4753)	13 545 (3766)	675 (353)	777 (482)
Theatre (including equipment)	7609 (4130)	3256 (1100)	4589 (1762)	5788 (1147)	6127 (1349)	7649 (2869)
Staff	5342 (3610)	1768 (1212)	2782 (1459)	3375 (899)	3642 (1059)	4836 (2251)
Blood products	351 (496)	69 (365)	121 (213)	2116 (3061)	2050 (3802)	2133 (996)
Total index procedure	45 147 (2923)	24 359 (8861)	37 406 (5953)	24 823 (5 266)	12 493 (5704)	15 394 (6598)
**Post-procedure and index costs**						
Blood products	2814 (3980)	49 (152)	276 (383)	365 (558)	159 (324)	1669 (1835)
Diagnostic interventions	1088 (994)	220 (304)	335 (286)	510 (517)	417 (505)	1255 (923)
Critical care bed days†	16 171 (18 860)	2929 (4348)	6695 (4809)	10 715 (7146)	15 765 (16 424)	59 540 (62 150)
Ward days	4169 (5306)	2807 (3549)	3726 (3173)	5452 (4209)	9797 (15 332)	61 076 (81 658)
Return to theatre events (*n*)	1	10	6	3	1	3
Return to theatre	840 (1189)	312 (1115)	1310 (2572)	158 (320)	52 (234)	3881 (5488)
Total post-procedure until discharge	25 083 (30 329)	6316 (6843)	12 362 (5619)	17 199 (11 259)	26 191 (27 351)	127 421 (152 054)
**Costs to NHS from index procedure up to discharge**						
Total costs	70 231 (27 406)	30 675 (11 920)	49 768 (9120)	42 023 (14 218)	48 151 (53 541)	142 816 (158 652)

All costs are reported in pounds Sterling (£) as mean ± standard deviation unless otherwise noted.

^*^Endovascular device for TEVAR patients surgical graft for OSR patients.

†Critical care bed days include intensive care unit and high-dependency unit.

Within the OSR group, those with hybrid surgical grafts (*n* = 13) had a mean (s.d.) procedure cost almost twice as much as those with standard thoracic repair, £24 854 (£8258) and £12 757 (£5678), respectively. The two patients with thoracoabdominal repair had slightly higher procedure costs of £15 394 (£6598) *versus* standard thoracic repair. Higher procedure costs for hybrid graft patients were driven by the high expense of the surgical graft £13 545 (£3766). Costs until discharge were much lower for the hybrid group £17 199 (£11 259) compared to standard repair £26 191 (£27 351). However, thoracoabdominal repair had very high costs until discharge recorded, £127 421 (£152 054), driven by high critical care and length of stay costs. Overall, hybrid costs were the lowest of the OSR subgroups £42 023 (£14 218), followed by standard thoracic repair £48 151 (£53 541), with thoracoabdominal aneurysm repair having a very large overall cost £142 816 (£158 652).

Due to small numbers, it is only appropriate to compare between descending thoracic aortic repair (TEVAR *n* = 97) and standard thoracic repair (OSR *n* = 20). Overall costs are higher in the standard thoracic repair OSR subgroup, with costs driven by critical care and ward days that occur in the post-procedure period. For descending thoracic aortic repair costs were driven by the high stent device costs that occur during the procedure.

### Twelve-month follow-up

Ninety-one TEVAR patients and 24 OSR patients had 12-month follow-up, with costs presented in *Table 4*. Mean (s.d.) follow-up costs were slightly higher for TEVAR £5206 (£11 585) compared to OSR £5039 (£11 994). Primary and secondary care costs in total were higher for OSR. Hospital readmissions costs were higher for TEVAR £1379 (£4738) compared to OSR £15 (£75), but re-intervention costs were higher for OSR £3745 (£12 013) compared to TEVAR £3036 (£10 802).

**Table 4:** Costs of NHS resource use from discharge to follow-up at 12 months for patients who underwent endovascular stent grafting (TEVAR) or open surgical repair (OSR)

Costs (£)	TEVAR (*n* = 91)	OSR (*n* = 24)
Primary care	288 (458)	538 (813)
Secondary care	503 (169)	741 (189)
Hospital readmissions events (*n*)	18	1
Hospital readmissions	1379 (4738)	15 (75)
Re-interventions events (*n*)	7	4
Re-interventions	3036 (10 802)	3 745 (12 013)
Total NHS cost at 12-month follow-up from discharge	5206 (11 585)	5039 (11 994)

All costs are reported in pounds Sterling (£) as mean (s.d.) unless otherwise noted.

### Subgroup analysis

Subgroup analysis was conducted to compare costs of follow-up that differed in terms of complexity within TEVAR and OSR (*Table 5*). Within the TEVAR cohort, those undergoing complex repair thoracoabdominal stent with fenestrated or branch grafting had higher costs from follow-up £5912 (£11 152) compared to TEVAR descending thoracic aortic repair £4 794 (£11 180), with aortic arch endovascular repair having a much lower follow-up cost £684 (£274). Primary care and secondary care costs were similar between the different TEVAR groups. No hospital admissions or re-interventions were recorded for the aortic arch patients. There were five hospital readmissions recorded for those undergoing complex thoracoabdominal TEVAR with a mean (s.d.) cost of £11 112 (£2038) per readmission and 13 hospital readmissions recorded for descending thoracic repair with a mean (s.d.) cost of £4466 (£4267) per admission. Overall, aneurysm-related hospital admissions costs were higher for the complex TEVAR group £5051 (£11 077) *versus* descending thoracic repair £915 (£2881). Only the descending thoracic repair group had re-interventions recorded with a mean (s.d.) cost of £39 467 (£8527) per reintervention, resulting in a mean (s.d.) cost of £3087 (£10 882) across the cohort.

**Table 5:** Subgroup analysis of costs of NHS resource use from discharge to follow-up at 12 months

Cost categories (£)	TEVAR (*n* = 91)			OSR (*n* = 24)		
	Aortic arch endovascular repair (*n* = 2)	Descending thoracic aortic repair (*n* = 78)	Complex repair thoracoabdominal stent with fenestrated or branch grafting (*n* = 11)	Hybrid grafts (*n* = 8)	Standard thoracic repair (*n* = 14)	Thoracoabdominal aneurysm repair (*n* = 2)
Primary care	126 (178)	291 (487)	331 (306)	450 (357)	666 (1017)	0 (0)
Secondary care	558 (96)	501 (178)	529 (103)	761 (240)	763 (137)	507 (239)
Hospital readmissions events (*n*)	0	13	5	0	1	0
Hospital readmissions	0 (0)	915 (2881)	5051 (11 077)	0 (0)	26 (98)	0 (0)
Re-interventions events (*n*)	0	7	0	4	0	0
Re-interventions	0 (0)	3087 (10 882)	0 (0)	11 234 (19 442)	0 (0)	0 (0)
Total NHS cost at 12-month follow-up from discharge	684 (274)	4794 (11 180)	5912 (11 152)	12 445 (19 406)	1455 (1002)	507 (239)

All costs are reported in pounds Sterling (£) as mean (s.d.) unless otherwise noted. TEVAR = endovascular stent graft; OSR = open surgical repair.

Within the OSR group, those with hybrid grafts (*n* = 8) had a much higher 12-month follow-up cost of £12 445 (£19 406) compared to £1455 (£1002) of those who underwent standard thoracic repair (*n* = 14). This higher cost was driven by re-interventions with the hybrid graft cohort recording four re-interventions with a mean (s.d.) cost of £22 468 (£31 639) per re-intervention, resulting in an mean (s.d.) cost of £11 234 (£19 442) across the hybrid graft group. There were no re-interventions recorded in the standard thoracic repair group. One aneurysm-related hospital admission was recorded in the standard thoracic repair group with a cost of £364, resulting in a mean (s.d.) cost to the group of £23 (£91). Twelve-month follow-up costs were low, £507 (£239) for the thoracoabdominal aneurysm repair (*n* = 2) subgroup, with only secondary care recorded.

Due to small numbers, it is only appropriate to compare subgroups between descending thoracic aortic repair (TEVAR *n* = 78) and standard thoracic repair (OSR *n* = 14). For these subgroups, TEVAR descending thoracic repair had a much higher mean 12-month cost compared to standard thoracic repair. Higher mean costs for descending thoracic repair were caused by higher recorded hospital admissions and re-interventions in this subgroup compared to standard thoracic repair.

## Discussion

Although costing is undertaken from a UK NHS perspective, the micro-costing methodology provides a detailed insight into the true resource inputs of providing OSR and TEVAR in routine clinical care. The micro-costing methodology defined as the ‘direct enumeration and costing of every input consumed in the treatment of a particular patient’^17^ involves a number of stages, the first being a detailed insight into the identification of all the resources involved in the provision of care. This study has relevance in international settings as the micro-costing approach used may help inform future accurate renumeration studies as resource inputs are unlikely to differ across different healthcare systems, particularly in terms of the surgical procedure itself. This is important because accurate information regarding the costs of surgical interventions is vital to inform policy and guidance^23^, suggesting these findings are transferable where national prices can be applied.

Estimated mean costs from index procedure up until 12-month follow-up are higher for OSR relative to TEVAR, which is largely driven by differences in costs up until discharge. The procedure cost for TEVAR accounts for over three-quarters of mean total cost until discharge, with these driven by the costs of the endovascular stent graft device used. While the costs of OSR procedures are lower, there are higher costs until discharge, driven by length of stay in critical care. Mean follow-up costs up at 12 months were higher for TEVAR, driven by re-interventions, although these were not enough to outweigh the higher in-hospital costs of OSR.

The NHS England tariff (reimbursement) for TEVAR and OSR procedures does not include the cost of critical care, which is determined on a trust-by-trust basis, or the cost of the endovascular stent graft, which is funded centrally^21^. For a meaningful comparison with NHS tariff, critical care and stent costs were removed from the ETTAA patient cost estimates. The NHS England tariff for TEVAR in 2018–19, the same price year that our cost analysis was conducted, was £7880 for an elective complex procedure and £7272 for an elective simple procedure. TEVAR patients in the ETTAA study had a mean (s.d.) cost of £9370 (£5584), which was higher than the tariff. Furthermore, estimated mean (s.d.) costs for patients undergoing OSR was £24 023 (£25 621), which was also significantly higher than the £15 722 tariff. These costing estimates therefore have significant implications for hospitals, suggesting provision of treatment may not be adequately compensated. Centralization of services means this burden will be on larger specialist units. There are non-financial benefits of providing these treatments and a societal need to provide a regional service, but these results suggest there will be little incentive to continue this work from a financial point of view and calls for reorganization of payments may be welcomed. At the time of the ETTAA study, stent costs were negotiated on a hospital-by-hospital basis with manufacturers, with stent costs included in this study collected directly from manufacturers. However, the NHS moving towards central purchasing^24^ of endovascular stent grafts may provide the opportunity to greatly reduce TEVAR procedure costs and make substantial cost savings. The development and introduction of more expensive and complex technology such as more sophisticated arch stent grafts should be carefully considered by health systems, particularly with high costs involved in these groups.

Previous studies conducted in the USA^25,26^ estimated costs of TEVAR and OSR for open elective repair of descending thoracic aortas in a single centre using the hospital’s accounting system found similar results regarding in-hospital cost drivers for each procedure. Endograft costs were a predictor of TEVAR costs with postoperative complications and length of stay being a predictor of OSR hospitalization costs. There is one other UK-based economic analysis that estimated costs of OSR and TEVAR from a consecutive series of 84 patients undergoing intervention on the descending aorta over a 13-year period using pre-, peri- and postoperative data from a single centre^27^. However, the procedure resources and associated costs were not micro-costed but based on a consensus regarding resource inputs with costs estimated at a ‘broad level’. Hospital costs were estimated from NHS reference costs and included staff time, consumables and length of stay. The findings of this study are like ours where OSR incurred higher costs relating to staff, consumables, transfusion and length of stay. However, they report that stent costs of TEVAR completely outweighed these higher costs with no difference in total costs reported overall (median (i.q.r.) OSR £15 045 (£9299–£27 571) and TEVAR £16 694 (£13 352–£21 729), *P* = 0.41). These median costs are also much lower than those identified in our study, even accounting for inflation, possibly due to a less-precise costing approach undertaken.

Most cost analyses of TAA repairs have only analysed hospital costs during the index hospitalization. Some of these studies have shown TEVAR to be less expensive due to shorter hospital stays with lower complication rates^25,28,29^, while others have shown no difference between open and endovascular repairs^27^. Other studies have reported overall lower hospitalization costs for TEVAR relative to OSR^30^. One study has evaluated costs beyond the initial hospital stay. Karimi *et al*.^25^ evaluated their TAA cost data in a 57-patient, single-centre cohort for 2 years postintervention. They found that in-hospital and at 2 years postintervention, TEVAR was the more cost-effective option. Gillen *et al*.^30^ conducted an in-hospital costing of both procedures and also assessed costs at 3 years utilizing a Monte Carlo simulation model and found that costs were also lower. However, except for Glade *et al*.^28^, who used data from three vascular centres in Amsterdam, most of these studies estimated in-hospital costs from a single-centre institution. These details of resource utilization and associated costs were estimated based on either a retrospective medical record review^28^ or individual organization’s financial accounting review^25,29^. No studies estimated costs from a health system perspective, rather than costs incurred by hospitals only. In addition, none of the studies to date adopted a detailed micro-costing exercise and presented details of costing methodologies that should be adopted when undertaking costing studies^31^, particularly in relation to annuitization of costs and discounting.

There are several limitations of this study. Micro-costing provides a more accurate method of resource-use assessment in economic analyses of surgical interventions^23^. Despite the in-depth cost analysis undertaken using a whole National Health Service costing perspective, the sample size of the population for OSR (*n* = 35) was small relative to TEVAR (*n* = 115). This was due to the subsample of the ETTAA population being eligible with no recorded contraindication to either procedure in order to ensure a less-biased comparison. It was identified that the majority of patients in the ETTAA study who had an OSR procedure were not eligible for TEVAR, with the reasons reported elsewhere^11^. However, the main focus of micro-costing as a methodology is precision regarding the assessment of the economic costs of a healthcare intervention^15^ and identifying key cost drivers. It has been highlighted that micro-costing studies vary widely in methodological and reporting quality, with a need to standardize methods and reporting of these studies and develop tools for their evaluation^32^. However, despite these debates in the health economics literature, sample sizes are not a research priority with a focus on accuracy and transparency. Given the detail and precision of our costing methodology, the cost estimates we present are likely to be representative of costs in routine clinical practice.

As reported previously^11^, it was clear that there were also differences in characteristics between the two surgical groups despite patients being eligible for both procedures. For example, TEVAR patients were older with smaller aneurysm size and more OSR patients had aneurysm extending into the aortic arch. Although the group included in this analysis are a subset of the larger ETTAA cohort study who were eligible for both OSR and TEVAR, there are still differences in anatomy between patients which may impact costs. Direct comparison between cohorts may result in possible bias as the sample sizes did not allow for control for potential confounding in costs. However, many of the resource inputs will be fixed in nature (for example, surgical equipment costs) and may not be strongly influenced by population characteristics or sample size.

The micro-costing methods and results presented in this study are of great value to help guide future research for the cost-effectiveness comparison of TEVAR *versus* OSR for treatment of distal arch/descending CTAA and are important to providers and decision makers for the purposes of developing guidelines. This study has identified and quantified the extent to which TEVAR procedure costs are driven by the high cost of endovascular stent graft and OSR procedure costs are driven by stay in critical care. Furthermore, this study has identified that NHS tariffs for TEVAR and OSR may be lower than the true cost of TEVAR and OSR procedures for the elective treatment of arch/descending thoracic aortic aneurysms, suggesting providers may not be adequately compensated.

## Collaborators

ETTAA Collaborative Group

Stephen Large, Linda Sharples, Luke Vale, Priya Sastry, Colin Bicknell, Carol Freeman, Andrew Cook, Yi-Da Chiu, Andrew McCarthy, Jo Gray, Peter McMeekin, S Rao Vallabhaneni, Nicky Watson, Dilupa Samarakoon, Thomas Devine, Tom Duffy, Victoria Hughes

## Supplementary Material

znad378_Supplementary_Data

## Data Availability

The data underlying this article cannot be shared publicly for the privacy of individuals that participated in the study. The data will be shared on reasonable request to the senior author J.G.

## Declaration of Helsinki

The authors confirm that the ETTAA study complies with the Declaration of Helsinki; that the West Midlands—South Birmingham Research Ethics Committee approved the research protocol and that informed consent was obtained from all participants.

## References

[1] Karthikesalingam A , Bahia SS, Patterson BO, Peach G, Vidal-Diez A, Ray KK et al The shortfall in long-term survival of patients with repaired thoracic or abdominal aortic aneurysms: retrospective case–control analysis of hospital episode statistics. Eur J Vasc Endovasc Surg 2013;46:533–541 24091096 10.1016/j.ejvs.2013.09.008

[2] Kuzmik GA , Sang AX, Elefteriades JA. Natural history of thoracic aortic aneurysms. J Vasc Surg 2012;56:565–571 22840907 10.1016/j.jvs.2012.04.053

[3] Desai ND , Burtch K, Moser W, Moeller P, Szeto WY, Pochettino A et al Long-term comparison of thoracic endovascular aortic repair (TEVAR) to open surgery for the treatment of thoracic aortic aneurysms. J Thorac Cardiovasc Surg 2012;144:604–611 22898503 10.1016/j.jtcvs.2012.05.049

[4] Kalteis M , Haller F, Artmann A, Ratzenböck M, Hartl P, Lugmayr H. Experience and outcomes after a decade of endovascular abdominal aortic aneurysm repair: a retrospective study from a community-based single center. Ann Vasc Surg 2012;26:330–337 22285344 10.1016/j.avsg.2011.06.012

[5] von Allmen RS , Anjum A, Powell JT. Outcomes after endovascular or open repair for degenerative descending thoracic aortic aneurysm using linked hospital data. Br J Surg 2014;101:1244–1251 25048981 10.1002/bjs.9568

[6] National Institute of Health and Care Excellence . Abdominal Aortic Anruysm: Diagnosis and Management (NICE Guideline NG156). London: NICE, 2019, 34–35

[7] Ross CILSMHS . The Reconfiguration of Clinical Services What is the Evidence? London: The King's Fund, 2014

[8] National Institute for Health and Care Excellence . Developing NICE Guidelines: The Manual. London: NICE, 2015, 128–157 26677490

[9] Knies S , Severens JL, Brouwer WBF. Integrating clinical and economic evidence in clinical guidelines: more needed than ever! J Eval Clin Pract 2019;25:561–564 29700903 10.1111/jep.12936 PMC6767471

[10] Sastry P , Hughes V, Hayes P, Vallabhaneni S, Sharples L, Thompson M et al The ETTAA study protocol: a UK-wide observational study of ‘effective treatments for thoracic aortic aneurysm’. BMJ Open 2015;5:e008147 10.1136/bmjopen-2015-008147 PMC4458682 26038360

[11] Sharples L , Sastry P, Freeman C, Gray J, McCarthy A, Chiu Y-D et al Endovascular stent grafting and open surgical replacement for chronic thoracic aortic aneurysms: a systematic review and prospective cohort study. Health Technol Assess (Rockv) 2022;26:1–166 10.3310/ABUT7744 35094747

[12] Husereau D , Drummond M, Augustovski F, de Bekker-Grob E, Briggs AH, Carswell C et al Consolidated health economic evaluation reporting standards 2022 (CHEERS 2022) statement: updated reporting guidance for health economic evaluations. J Med Econ 2022;25:1–7 10.1080/13696998.2021.2014721 35012427

[13] von Elm E , Altman DG, Egger M, Pocock SJ, Gøtzsche PC, Vandenbroucke JP. The strengthening the reporting of observational studies in epidemiology (STROBE) statement: guidelines for reporting observational studies. Lancet 2007;370:1453–1457 18064739 10.1016/S0140-6736(07)61602-X

[14] Mercier G , Naro G. Costing hospital surgery services: the method matters. PLoS One 2014;9:e97290 24817167 10.1371/journal.pone.0097290 PMC4016301

[15] Polsky D , Glick H. Costing and cost analysis in randomized controlled trials: *caveat emptor*. Pharmacoeconomics 2009;27:179–188 19354338 10.2165/00019053-200927030-00001 PMC2971527

[16] Drummond MF , Sculpher MJ, Claxton K, Stoddard GL, Torrance GW. Methods for the Economic Evaluation of Health Care Programmes (4th edn). Oxford, UK: Oxford University Press, 2015

[17] Gold MR , Siegel JE, Russel LB, Weinstein MC. Cost-Effectiveness in Health and Medicine. New York, NY: Oxford University Press Inc, 1996

[18] National Institute for Health and Care Excellence . Guide to the Methods of Technology Appraisal. London, 2018 27905712

[19] Walker D , Kumaranayake L. Allowing for differential timing in cost analyses: discounting and annualization. Health Policy Plan 2002;17:112–118 11861593 10.1093/heapol/17.1.112

[20] Sharples L , Sastry P, Freeman C et al Endovascular Stent Grafting and Open Surgical Replacement for Chronic Thoracic Aortic Aneurysms: A Systematic Review and Prospective Cohort Study: Health Technology Assessment, 2022:76–79 10.3310/ABUT7744 35094747

[21] Department of Health and Social Care . NHS Reference Costs 2018 to 2019. London: Department of Health and Social Care, 2019

[22] Curtice L , Burns A. Unit Costs of Health and Social Care 2019. Canterbury: Unit PSSR, University of Kent, 2019

[23] Potter S , Davies C, Davies G, Rice C, Hollingworth W. The use of micro-costing in economic analyses of surgical interventions: a systematic review. Health Econ Rev 2020;10:3 31997021 10.1186/s13561-020-0260-8 PMC6990532

[24] National supply system for high-cost tariff-excluded devices. https://www.england.nhs.uk/commissioning/spec-services/key-docs/medical-devices/ (accessed 10 October 2021)

[25] Karimi A , Walker KL, Martin TD, Hess PJ, Klodell CT, Feezor RJ et al Midterm cost and effectiveness of thoracic endovascular aortic repair *versus* open repair. Ann Thorac Surg 2012;93:473–479 22197617 10.1016/j.athoracsur.2011.10.016

[26] Walker KL , Lipori P, Lee WA, Beaver TM. Cost of thoracic endovascular aortic repair *versus* open repair and implications for the US health care system. J Thorac Cardiovasc Surg 2010;139:231–232 19691999 10.1016/j.jtcvs.2009.07.020

[27] Narayan P , Wong A, Davies I, Angelini GD, Bryan AJ, Wilde P et al Thoracic endovascular repair *versus* open surgical repair—which is the more cost-effective intervention for descending thoracic aortic pathologies? Eur J Cardiothorac Surg 2011;40:869–874 21353586 10.1016/j.ejcts.2011.01.010

[28] Glade GJ , Vahl AC, Wisselink W, Linsen MAM, Balm R. Mid-term survival and costs of treatment of patients with descending thoracic aortic aneurysms; endovascular vs. open repair: a case–control study. Eur J Vasc Endovasc Surg 2005;29:28–34 15570268 10.1016/j.ejvs.2004.10.003

[29] Schuster I , Dorfmeister M, Scheuter-Mlaker S, Gottardi R, Hoebartner M, Roedler S et al Endovascular and conventional treatment of thoracic aortic aneurysms: a comparison of costs. Ann Thorac Surg 2009;87:1801–1805.e1806 19463598 10.1016/j.athoracsur.2009.02.099

[30] Gillen JR , Schaheen BW, Yount KW, Cherry KJ, Kern JA, Kron IL et al Cost analysis of endovascular *versus* open repair in the treatment of thoracic aortic aneurysms. J Vasc Surg 2015;61:596–603 25449008 10.1016/j.jvs.2014.09.009 PMC4344903

[31] Ramsey SD , Willke RJ, Glick H, Reed SD, Augustovski F, Jonsson B et al Cost-effectiveness analysis alongside clinical trials II—an ISPOR good research practices task force report. Value Health 2015;18:161–172 25773551 10.1016/j.jval.2015.02.001

[32] Xu X , Lazar CM, Ruger JP. Micro-costing in health and medicine: a critical appraisal. Health Econ Rev 2021;11:1 33404857 10.1186/s13561-020-00298-5 PMC7789519

