# Supplementary material for: Costs of endovascular and open repair of thoracic aortic aneurysms
Source: Br J Surg. 2023 Dec 13;111(1):znad378. doi: 10.1093/bjs/znad378 (PMC10763539; doi:10.1093/bjs/znad378)
Supplement: znad378_Supplementary_Data [file znad378_supplementary_data.zip › Supplementary-Material.docx]

**Costs of endovascular and open repair of thoracic aortic aneurysms**

**Authors**

Joanne Gray^1^, Andrew McCarthy^1, *^, Dilupa Samarakoon^1^, Peter McMeekin^1^, Linda Sharples^2^, Priya Sastry^3^, Paul Crawshaw^4^, Colin Bicknell^5^, and on behalf of the ETTAA Collaborative Group

**Institutions**

1 Faculty of Health and Life Sciences, Northumbria University, Newcastle Upon Tyne, UK

2 Department of Medical Statistics, London School of Hygiene and Tropical Medicine, London, UK

3 Department of Cardiac Surgery, John Radcliffe Hospital, Oxford University Hospitals, UK

4 School of Social Sciences, Humanities and Law, Teesside University, UK

5 Department of Surgery and Cancer, Imperial College, London, UK and Imperial Vascular Unit, Imperial Healthcare NHS Trust, London, UK

**Corresponding author.** **Corresponding Author:** Andrew McCarthy; telephone: 0191 215 6718, Email: [andrew2.mccarthy@northumbria.ac.uk](mailto:andrew2.mccarthy@northumbria.ac.uk)  **ORCID ID**; 0000-0002-3385-6302

**Collaborators**

ETTAA Collaborative Group

Stephen Large, Linda Sharples, Luke Vale, Priya Sastry, Colin Bicknell, Carol Freeman, Andrew Cook, Yi-Da Chiu, Andrew McCarthy, Jo Gray, Peter McMeekin, S Rao Vallabhaneni, Nicky Watson, Dilupa Samarakoon, Thomas Devine, Tom Duffy, Victoria Hughes

**Supplementary Materials – Index**

| **Supplementary Figures and Tables** |  |
| --- | --- |
| Figure S1 | *Page 2* |
| Table S1 | *Page 3* |
| Table S2 | *Page 13* |
| Table S3 | *Page 15* |
| Table S4 | *Page 17* |
| Table S5 | *Page 25* |
| Table S6 | *Page 26* |
| Table S7 | *Page 27* |
| Table S8 | *Page 28* |

**Figure S1: Patient Flow Diagram**

**
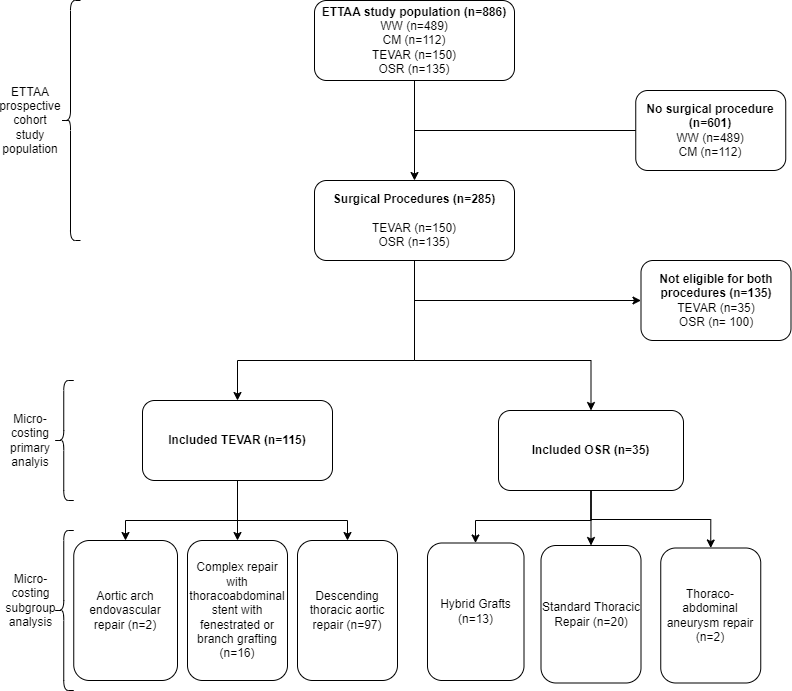
**

**Table S1: Mean resource use of index procedure**

| **Resource or unit intervention** | **Unit** | **Mean usage in standard TEVAR** | **Mean usage in standard OSR** | **Resource Source** |
| --- | --- | --- | --- | --- |
| **Fixed costs**  ***Theatre usage overheads*** | | | | |
| Operating room | Average theatre duration | 4 hours 56 minutes | 8 hours 53 minutes | ETTAA study procedure CRF form |
| Operating room with C arm | Average theatre duration | 4 hours 52 minutes | N/A | ETTAA study procedure CRF form |
| Catheter lab | Average theatre duration | 4 hours 5mins | N/A | ETTAA study procedure CRF form |
| Hybrid theatre | Average theatre duration | 3 hours 19 minutes | N/A | ETTAA study procedure CRF form |
| Interventional radiology equipment | Per hour | 1 | N/A | Imperial College Healthcare NHS Trust based on personal communication with Colin Bicknell between Jan-June 2020 |
| ***Capital Equipment*** | | | | |
| Cooling head jacket | Per procedure | N/A | 1 | Royal Papworth Hospital NHS Trust based on personal communication with Rosie Thornton between Nov 2019-20 |
| Basic vascular tray | Per procedure | 2 | N/A | Imperial College Healthcare NHS Trust based on personal communication with Colin Bicknell between Jan-June 2020 |
| Cardiac major tray | Per procedure | N/A | 1 | Royal Papworth Hospital NHS Trust based on personal communication with Rosie Thornton between Nov 2019-20 |
| Diathermy console | Per procedure | 1 | 1 | Royal Papworth Hospital NHS Trust based on personal communication with Rosie Thornton between Nov 2019-20 |
| Sternal saw | Per procedure | N/A | 1 | Royal Papworth Hospital NHS Trust based on personal communication with Rosie Thornton between Nov 2019-20 |
| Defibrillator paddles | Per procedure | N/A | 1 | Royal Papworth Hospital NHS Trust based on personal communication with Rosie Thornton between Nov 2019-20 |
| Cell saver machine | Per procedure | N/A | 1 | Royal Papworth Hospital NHS Trust based on personal communication with Rosie Thornton between Nov 2019-20 |
| Sternal retractors | Per procedure | N/A | 1 | Royal Papworth Hospital NHS Trust based on personal communication with Rosie Thornton between Nov 2019-20 |
| By-pass machine | Per procedure | N/A | 1 | Royal Papworth Hospital NHS Trust based on personal communication with Rosie Thornton between Nov 2019-20 |
| Bair hugger machine | Per procedure | N/A | 1 | Royal Papworth Hospital NHS Trust based on personal communication with Rosie Thornton between Nov 2019-20 |
| Rapid transfuser/fluid warmer | Per procedure | N/A | 1 | Royal Papworth Hospital NHS Trust based on personal communication with Rosie Thornton between Nov 2019-20 |
| Suction machine | Per procedure | 1 | 1 | Imperial College Healthcare NHS Trust based on personal communication with Colin Bicknell between Jan-June 2020  Royal Papworth Hospital NHS Trust based on personal communication with Rosie Thornton between Nov 2019-20 |
| Injection pump | Per procedure | 1 | 4 | Imperial College Healthcare NHS Trust based on personal communication with Colin Bicknell between Jan-June 2020  Royal Papworth Hospital NHS Trust based on personal communication with Rosie Thornton between Nov 2019-20 |
| **Staff** | | | | |
| Consultant surgeon | Per hour | 1 | 1 | Imperial College Healthcare NHS Trust based on personal communication with Colin Bicknell between Jan-June 2020  Royal Papworth Hospital NHS Trust based on personal communication with Rosie Thornton between Nov 2019-20 |
| Assistant surgeon | Per hour | 1 | 1 | Imperial College Healthcare NHS Trust based on personal communication with Colin Bicknell between Jan-June 2020  Royal Papworth Hospital NHS Trust based on personal communication with Rosie Thornton between Nov 2019-20 |
| Consultant anaesthetist | Per hour | Included in theatre cost except catheter lab | Included in theatre cost | Imperial College Healthcare NHS Trust based on personal communication with Colin Bicknell between Jan-June 2020 |
| Anaesthetist registrar | Per hour | Included in theatre cost except catheter lab | Included in theatre cost | Imperial College Healthcare NHS Trust based on personal communication with Colin Bicknell between Jan-June 2020 |
| Consultant radiologist | Per hour | 1 | N/A | Imperial College Healthcare NHS Trust based on personal communication with Colin Bicknell between Jan-June 2020 |
| Assistant/registrar radiologist | Per hour | 1 | N/A | Imperial College Healthcare NHS Trust based on personal communication with Colin Bicknell between Jan-June 2020 |
| Radiographer | Per hour | 1 | N/A | Imperial College Healthcare NHS Trust based on personal communication with Colin Bicknell between Jan-June 2020 |
| Surgical care practitioner | Per hour | N/A | 1 | Royal Papworth Hospital NHS Trust based on personal communication with Rosie Thornton between Nov 2019-20 |
| Anaesthetic nurse | Per hour | 1 | 1 | Royal Papworth Hospital NHS Trust based on personal communication with Rosie Thornton between Nov 2019-20  Imperial College Healthcare NHS Trust based on personal communication with Colin Bicknell between Jan-June 2020 |
| Scrub nurse (table) | Per hour | 1 | 1 | Imperial College Healthcare NHS Trust based on personal communication with Colin Bicknell between Jan-June 2020  Royal Papworth Hospital NHS Trust based on personal communication with Rosie Thornton between Nov 2019-20 |
| Scrub nurse (floor) | Per hour | 1 | 1 | Imperial College Healthcare NHS Trust based on personal communication with Colin Bicknell between Jan-June 2020  Royal Papworth Hospital NHS Trust based on personal communication with Rosie Thornton between Nov 2019-20 |
| Healthcare assistant | Per hour | 1 | N/A | Imperial College Healthcare NHS Trust based on personal communication with Colin Bicknell between Jan-June 2020 |
| Perfusionist | Per hour | N/A | 1 | Royal Papworth Hospital NHS Trust based on personal communication with Rosie Thornton between Nov 2019-20 |
| **Consumables** | | | | |
| Central lines | Per item | 1 | 1 | Imperial College Healthcare NHS Trust based on personal communication with Colin Bicknell between Jan-June 2020  Royal Papworth Hospital NHS Trust based on personal communication with Rosie Thornton between Nov 2019-20 |
| Arterial lines | Per item | 1 | 1 | Imperial College Healthcare NHS Trust based on personal communication with Colin Bicknell between Jan-June 2020  Royal Papworth Hospital NHS Trust based on personal communication with Rosie Thornton between Nov 2019-20 |
| Nasopharyngeal probe | Per item | N/A | 1 | Royal Papworth Hospital NHS Trust based on personal communication with Rosie Thornton between Nov 2019-20 |
| Urinary catheter | Per item | 1 | 1 | Imperial College Healthcare NHS Trust based on personal communication with Colin Bicknell between Jan-June 2020  Royal Papworth Hospital NHS Trust based on personal communication with Rosie Thornton between Nov 2019-20 |
| Diathermy pad | Per item | N/A | 1 | Royal Papworth Hospital NHS Trust based on personal communication with Rosie Thornton between Nov 2019-20 |
| Swabs | Per item | 20 | 60 | Imperial College Healthcare NHS Trust based on personal communication with Colin Bicknell between Jan-June 2020  Royal Papworth Hospital NHS Trust based on personal communication with Rosie Thornton between Nov 2019-20 |
| Size 15 knife blade | Per item | N/A | 1 | Royal Papworth Hospital NHS Trust based on personal communication with Rosie Thornton between Nov 2019-20 |
| Size 10 knife blade | Per item | N/A | 1 | Royal Papworth Hospital NHS Trust based on personal communication with Rosie Thornton between Nov 2019-20 |
| Cell saver tubing and fluids | Per item | N/A | 1 | Royal Papworth Hospital NHS Trust based on personal communication with Rosie Thornton between Nov 2019-20 |
| Side towels | Per item | N/A | 2 | Royal Papworth Hospital NHS Trust based on personal communication with Rosie Thornton between Nov 2019-20 |
| Vicryl stay suture | Per item | N/A | 1 | Royal Papworth Hospital NHS Trust based on personal communication with Rosie Thornton between Nov 2019-20 |
| Cannulae (all sorts) | Per item | N/A | 4 | Royal Papworth Hospital NHS Trust based on personal communication with Rosie Thornton between Nov 2019-20 |
| Axillary cannular | Per item | N/A | 1 | Royal Papworth Hospital NHS Trust based on personal communication with Rosie Thornton between Nov 2019-20 |
| 4/0 prolene | Per item | N/A | 10 | Royal Papworth Hospital NHS Trust based on personal communication with Rosie Thornton between Nov 2019-20 |
| 5/0 prolene | Per item | 3 | N/A | Imperial College Healthcare NHS Trust based on personal communication with Colin Bicknell between Jan-June 2020 |
| 3/0 prolene | Per item | N/A | 5 | Royal Papworth Hospital NHS Trust based on personal communication with Rosie Thornton between Nov 2019-20 |
| Maxalon | Per item | N/A | 1 | Royal Papworth Hospital NHS Trust based on personal communication with Rosie Thornton between Nov 2019-20 |
| Monocryl suture | Per item | 1 | N/A | Imperial College Healthcare NHS Trust based on personal communication with Colin Bicknell between Jan-June 2020 |
| 2/0 vicryl | Per item | 2 | 2 | Imperial College Healthcare NHS Trust based on personal communication with Colin Bicknell between Jan-June 2020  Royal Papworth Hospital NHS Trust based on personal communication with Rosie Thornton between Nov 2019-20 |
| By-pass circuit disposable bits | Per item | N/A | 1 | Royal Papworth Hospital NHS Trust based on personal communication with Rosie Thornton between Nov 2019-20 |
| Teflon pledgets/strips | Per item | N/A | 10 | Royal Papworth Hospital NHS Trust based on personal communication with Rosie Thornton between Nov 2019-20 |
| Bair hugger blanket | Per item | N/A | 1 | Royal Papworth Hospital NHS Trust based on personal communication with Rosie Thornton between Nov 2019-20 |
| Haemostatic adjuncts | Per item | N/A | 1 | Royal Papworth Hospital NHS Trust based on personal communication with Rosie Thornton between Nov 2019-20 |
| Sternal wires | Per item | N/A | 3 | Royal Papworth Hospital NHS Trust based on personal communication with Rosie Thornton between Nov 2019-20 |
| Biosyn | Per item | N/A | 1 | Royal Papworth Hospital NHS Trust based on personal communication with Rosie Thornton between Nov 2019-20 |
| Dressings | Per item | N/A | 2 | Royal Papworth Hospital NHS Trust based on personal communication with Rosie Thornton between Nov 2019-20 |
| Argyl drain | Per item | N/A | 2 | Royal Papworth Hospital NHS Trust based on personal communication with Rosie Thornton between Nov 2019-20 |
| Redivac drain | Per item | N/A | 1 | Royal Papworth Hospital NHS Trust based on personal communication with Rosie Thornton between Nov 2019-20 |
| Drain sutures braided nylon | Per item | N/A | 4 | Royal Papworth Hospital NHS Trust based on personal communication with Rosie Thornton between Nov 2019-20 |
| Spinal drain | Per item | 1 | N/A | Imperial College Healthcare NHS Trust based on personal communication with Colin Bicknell between Jan-June 2020 |
| Suction tube | Per item | 2 | N/A | Imperial College Healthcare NHS Trust based on personal communication with Colin Bicknell between Jan-June 2020 |
| Sheath | Per item | 2 | N/A | Imperial College Healthcare NHS Trust based on personal communication with Colin Bicknell between Jan-June 2020 |
| Angiography hollow needles | Per item | 1 | N/A | Imperial College Healthcare NHS Trust based on personal communication with Colin Bicknell between Jan-June 2020 |
| Pigtail catheter | Per item | 1 | N/A | Imperial College Healthcare NHS Trust based on personal communication with Colin Bicknell between Jan-June 2020 |
| J wire | Per item | 1 | N/A | Imperial College Healthcare NHS Trust based on personal communication with Colin Bicknell between Jan-June 2020 |
| Terumo wire | Per item | 1 | N/A | Imperial College Healthcare NHS Trust based on personal communication with Colin Bicknell between Jan-June 2020 |
| Super stiff Miere wire | Per item | 2 | N/A | Imperial College Healthcare NHS Trust based on personal communication with Colin Bicknell between Jan-June 2020 |
| Extension for injection pump | Per item | 1 | N/A | Imperial College Healthcare NHS Trust based on personal communication with Colin Bicknell between Jan-June 2020 |
| Iodinated contrast | Per item | 1 | N/A | Imperial College Healthcare NHS Trust based on personal communication with Colin Bicknell between Jan-June 2020 |
| Injection pump contract syringe | Per item | 1 | N/A | Imperial College Healthcare NHS Trust based on personal communication with Colin Bicknell between Jan-June 2020 |
| Measuring pigtail catheter | Per item | 1 | N/A | Imperial College Healthcare NHS Trust based on personal communication with Colin Bicknell between Jan-June 2020 |
| 12F Sheath | Per item | 2 | N/A | Imperial College Healthcare NHS Trust based on personal communication with Colin Bicknell between Jan-June 2020 |
| Saline | Per item | 2 | N/A | Imperial College Healthcare NHS Trust based on personal communication with Colin Bicknell between Jan-June 2020 |
| Sterile bowls for coiling wires | Per item | 1 | N/A | Imperial College Healthcare NHS Trust based on personal communication with Colin Bicknell between Jan-June 2020 |
| Moulding balloon | Per item | 1 | N/A | Imperial College Healthcare NHS Trust based on personal communication with Colin Bicknell between Jan-June 2020 |
| Syringe 20ml | Per item | 2 | N/A | Imperial College Healthcare NHS Trust based on personal communication with Colin Bicknell between Jan-June 2020 |
| Diathermy forceps | Per item | 1 | N/A | Imperial College Healthcare NHS Trust based on personal communication with Colin Bicknell between Jan-June 2020 |
| Blade | Per item | 1 | N/A | Imperial College Healthcare NHS Trust based on personal communication with Colin Bicknell between Jan-June 2020 |
| Surgicel | Per item | 1 | N/A | Imperial College Healthcare NHS Trust based on personal communication with Colin Bicknell between Jan-June 2020 |
| ***Blood products*** | | | | |
| Heparin | Per 1000 units/1ml solution | N/A | 0 | ETTAA study procedure CRF form |
| Protamine | Per sulfate 50mg/5ml | N/A | 0 | ETTAA study procedure CRF form |
| Standard Red cells | Per unit | 0.46 | 5.06 | ETTAA study procedure CRF form |
| Platelets, Pooled | Per unit | 0.07 | 1.63 | ETTAA study procedure CRF form |
| FFP | Per unit (275ml) | 0.10 | 3.51 | ETTAA study procedure CRF form |
| Cryoprecipitate, Pooled | Per unit (200ml) | 0.03 | 2.26 | ETTAA study procedure CRF form |
| Octaplex | Per unit (500iu) | N/A | 2 | ETTAA study procedure CRF form |
| Beriplex | Per unit (500iu) | N/A | 0.06 | ETTAA study procedure CRF form |
| Fibrinogen | Per unit (1g) | N/A | 0.31 | ETTAA study procedure CRF form |
| Albumin | Per unit (100ml of 20%) | N/A | 0 | ETTAA study procedure CRF form |
| Novo7 | Per unit (2mg) | N/A | 0.29 | ETTAA study procedure CRF form |

**Table S2: Mean resource use post operatively until hospital discharge including return to theatre**

| Resource or intervention | Unit | Mean usage  in standard TEVAR | Mean usage  in standard OSR | Resource source |
| --- | --- | --- | --- | --- |
| **Type of stay** | | | | |
| ICU | Per day | 1.61 | 13.05 | ETTAA study, Post-Procedure and Discharge CRF form |
| HDU | Per day | 0.90 | 1.73 | ETTAA study, Post-Procedure and Discharge CRF form |
| Ward | Per day | 5.89 | 8.77 | ETTAA study, Post-Procedure and Discharge CRF form |
| Ward after transfer | Per day | 0.85 | 26.64 | ETTAA study, Post-Procedure and Discharge CRF form |
| **Blood products** | | | | |
| Standard red blood cells | Per unit | 0.33 | 1.64 | ETTAA study, Post-Procedure and Discharge CRF form |
| Platelets, Pooled | Per unit | 0.03 | 0.27 | ETTAA study, Post-Procedure and Discharge CRF form |
| FFP | Per unit (275ml) | 0.02 | 0.59 | ETTAA study, Post-Procedure and Discharge CRF form |
| Cryoprecipitate, Pooled | Per unit (200ml) | 0.00 | 0.09 | ETTAA study, Post-Procedure and Discharge CRF form |
| Albumin | Per unit | 0 | 0.09 | ETTAA study, Post-Procedure and Discharge CRF form |
| octuplex | Per unit | 0 | 0.06 | ETTAA study, Post-Procedure and Discharge CRF form |
| Plasmalyte | Per unit | 0.01 | 0 | ETTAA study, Post-Procedure and Discharge CRF form |
| **Imaging** | | | | |
| CT | Per investigation | 0.79 | 1.18 | ETTAA study, Post-Procedure and Discharge CRF form |
| MRI | Per investigation | 0.15 | 1.18 | ETTAA study, Post-Procedure and Discharge CRF form |
| X-ray (plain films) | Per investigation | 0.94 | 7.41 | ETTAA study, Post-Procedure and Discharge CRF form |
| TOE | Per investigation | 0.03 | 0.09 | ETTAA study, Post-Procedure and Discharge CRF form |
| TTE | Per investigation | 0.10 | 0.27 | ETTAA study, Post-Procedure and Discharge CRF form |
| Angiograph | Per investigation | 0.10 | 0.03 | ETTAA study, Post-Procedure and Discharge CRF form |
| Ultrasound | Per investigation | 0.19 | 0.20 | ETTAA study, Post-Procedure and Discharge CRF form |
| flouroscopy | Per investigation | 0 | 0.03 | ETTAA study, Post-Procedure and Discharge CRF form |
| Renogram | Per investigation | 0 | 0.03 | ETTAA study, Post-Procedure and Discharge CRF form |
| Echocardiogram | Per investigation | 0.02 | 0 | ETTAA study, Post-Procedure and Discharge CRF form |
| **Return to Theatre** | | | | |
| Return to Theatre | Per event | 0.11 | 0.18 | ETTAA study, Return to theatre CRF form |

**Table S3: Mean resource use of healthcare by follow-up including hospital readmissions and additional procedure**

| **Follow up - 1 month:** | | | |
| --- | --- | --- | --- |
|  |  | **TEVAR** | **OSR** |
| **Resource or intervention** | **Unit** | **Mean usage** | **Mean usage** |
| Nurse Visits | Per visit | 0.62 | 0.3 |
| Nurse Home Visits | Per visit | 0.57 | 0.4 |
| GP Visits | Per visit | 0.7 | 0.4 |
| GP home Visits | Per visit | 0.13 | 0.1 |
| Physio Visits | Per visit | 0.02 | 0 |
| A&E visits | Per visit | 0.16 | 0.2 |
| CT scans | Per visit | 1 | 0 |
| Outpatient Appointments Vascular Surgery (Consultant led) | Per visit | 1 | 0 |
| Outpatient Appointments Cardiothoracic Surgery (Consultant led) | Per visit | 0 | 0 |
| Additional procedures | Per event | 0.11 | 0 |
| Hospital Admissions | Per event | 0.07 | 0 |
| **Follow up - 3 month:** | | | |
|  |  | **TEVAR** | **OSR** |
| **Resource or intervention** | **Unit** | **Mean usage** | **Mean usage** |
| Nurse Visits | Per visit | 1.63 | 0.5 |
| Nurse Home Visits | Per visit | 0.17 | 0.08 |
| GP Visits | Per visit | 0.92 | 1.25 |
| GP home Visits | Per visit | 0.06 | 0 |
| Physio Visits | Per visit | 0.06 | 0.92 |
| A&E visits | Per visit | 0.08 | 0.25 |
| CT scans | Per visit | 0 | 0 |
| Outpatient Appointments Vascular Surgery (Consultant led) | Per visit | 0 | 0 |
| Outpatient Appointments Cardiothoracic Surgery (Consultant led) | Per visit | 0 | 0 |
| Additional procedures | Per event | 0.06 | 0 |
| Hospital Admissions | Per event | 0.02 | 0 |
| **Follow up - 6 month:** | | | |
|  |  | **TEVAR** | **OSR** |
| **Resource or intervention** | **Unit** | **Mean usage** | **Mean usage** |
| Nurse Visits | Per visit | 1.26 | 0.81 |
| Nurse Home Visits | Per visit | 0.26 | 3.5 |
| GP Visits | Per visit | 1.39 | 1.5 |
| GP home Visits | Per visit | 0.09 | 0 |
| Physio Visits | Per visit | 0.33 | 0.75 |
| A&E visits | Per visit | 0.09 | 0.13 |
| CT scans | Per visit | 0 | 1 |
| Outpatient Appointments Vascular Surgery (Consultant led) | Per visit | 0 | 0 |
| Outpatient Appointments Cardiothoracic Surgery (Consultant led) | Per visit | 0 | 1 |
| Additional procedures | Per event | 0.03 | 0 |
| Hospital Admissions | Per event | 0.01 | 0 |
| **Follow up - 12 month:** | | | |
|  |  | **TEVAR** | **OSR** |
| **Resource or intervention** | **Unit** | **Mean usage** | **Mean usage** |
| Nurse Visits | Per visit | 1.45 | 1 |
| Nurse Home Visits | Per visit | 2.38 | 12.15 |
| GP Visits | Per visit | 1.89 | 2.15 |
| GP home Visits | Per visit | 0.05 | 0.15 |
| Physio Visits | Per visit | 0.6 | 1.85 |
| A&E visits | Per visit | 0.25 | 0.15 |
| CT scans | Per visit | 1 | 1 |
| Outpatient Appointments Vascular Surgery (Consultant led) | Per visit | 1 | 0 |
| Outpatient Appointments Cardiothoracic Surgery (Consultant led) | Per visit | 0 | 1 |
| Additional procedures | Per event | 0.03 | 0 |
| Hospital Admissions | Per event | 0.02 | 0 |

**Table S4: Unit costs of resources and interventions primary procedure and return to theatre**

| **Resource or unit intervention** | **OSR** | **TEVAR** | **Cost (£)** | **Cost Source** | | |
| --- | --- | --- | --- | --- | --- | --- |
| **Fixed Costs** | | | | | | |
| Operating room for OSR | Yes | n/a | 518^ | Imperial College Healthcare NHS Trust based on personal communication with Colin Bicknell between Jan-June 2020 | | |
| Operating room for TEVAR* | n/a | Yes | 550.08*^ | Imperial College Healthcare NHS Trust based on personal communication with Colin Bicknell between Jan-June 2020 | | |
| Operating room with C arm* | n/a | Yes | 550.08*^ | Imperial College Healthcare NHS Trust based on personal communication with Colin Bicknell between Jan-June 2020 | | |
| Catheter laboratory | n/a | Yes | 252.08* | Royal Papworth Hospital NHS Trust (based on expert opinion) | | |
| Hybrid theatre | n/a | Yes | 550.08*^ | Imperial College Healthcare NHS Trust based on personal communication with Colin Bicknell between Jan-June 2020 | | |
| *includes interventional radiology equipment  ^consultant anaesthetist + anaesthetic registrar costs included | | | | | | |
| Interventional radiology (IR) equipment |  | Yes | 32.08 | Imperial College Healthcare NHS Trust based on personal communication with Colin Bicknell between Jan-June 2020 | | |
| **Capital equipment costs** | | | | | | |
| **Resource or unit intervention** | **OSR** | **TEVAR** | **Capital Cost (£)** | **Annualised Cost (£)** | **Cost (£) per operating session (253 days)** | **Cost Source** |
| Cooling head jacket | Yes | n/a | £12,000  (5 years) | £2,657 | £11 | Capital cost from Royal Papworth Hospital NHS Trust based on personal communication with Rosie Thornton between Nov 2019-20 |
| Basic vascular tray | n/a | Yes | £7000  (10 years) | £841.65 | £3.33 | Capital cost from Imperial College Healthcare NHS Trust based on personal communication with Colin Bicknell between Jan-June 2020 |
| Diathermy console | Yes | Yes | 9 000  (5 years) | 1 993 | 7.88 | Capital cost from Royal Papworth Hospital NHS Trust based on personal communication with Rosie Thornton between Nov 2019-20 |
| Sternal saw | Yes | n/a | 7 500  (5 years) | 1 661 | 6.57 | Capital cost from Royal Papworth Hospital NHS Trust based on personal communication with Rosie Thornton between Nov 2019-20 |
| Cell saver machine | Yes | n/a | 5 000  (5 years) | 1 107 | 4.38 | Capital cost from Royal Papworth Hospital NHS Trust based on personal communication with Rosie Thornton between Nov 2019-20 |
| Sternal retractors | Yes | n/a | 6 900  (5 years) | 1 528 | 6.04 | Capital cost from Royal Papworth Hospital NHS Trust based on personal communication with Rosie Thornton between Nov 2019-20 |
| By-pass machine | Yes | n/a | 10 500  (5 years) | 2 325 | 9.20 | Capital cost from Royal Papworth Hospital NHS Trust based on personal communication with Rosie Thornton between Nov 2019-20 |
| Bair hugger machine | Yes | n/a | 3 750  (1 year) | 3 880 | 15.35 | Capital cost from Royal Papworth Hospital NHS Trust based on personal communication with Rosie Thornton between Nov 2019-20 |
| Rapid transfuser/fluid warmer | Yes | n/a | 15 000  (5 years) | 3 222 | 13.13 | Capital cost from Royal Papworth Hospital NHS Trust based on personal communication with Rosie Thornton between Nov 2019-20 |
| Suction machine | n/a | Yes | 500  (5 years) | 111 | 0.44 | Capital cost from Imperial College Healthcare NHS Trust based on personal communication with Colin Bicknell between Jan-June 2020 |
| Injection pump | n/a | Yes | 2 000  (5 years) | 443 | 1.75 | Capital cost from Imperial College Healthcare NHS Trust based on personal communication with Colin Bicknell between Jan-June 2020 |
| Defibrillator paddles | Yes | n/a | 3 | | Capital cost from Royal Papworth Hospital NHS Trust based on personal communication with Rosie Thornton between Nov 2019-20 | |
| Cardiac major tray | Yes | n/a | 50 | | Capital cost from Royal Papworth Hospital NHS Trust based on personal communication with Rosie Thornton between Nov 2019-20 | |
| **Staff costs** | | | | | | |
| **Resource or unit intervention** | **OSR** | **TEVAR** | **Cost (£)** | | **Cost Source** | |
| Consultant surgeon | Yes | Yes | 109 | | PSSRU 2018/2019 based on “Consultant: surgical” | |
| Assistant surgeon | Yes | Yes | 47 | | PSSRU 2018/2019 based on “Registrar” | |
| Consultant anaesthetist  *(included in theatre cost, except cath lab)* | Yes | Yes | 109 | | PSSRU 2018/2019 based on “Consultant: medical” | |
| Anaesthetist registrar  *(included in theatre cost, except cath lab)* | Yes | Yes | 47 | | PSSRU 2018/2019 based on “Registrar” | |
| Consultant radiologist | n/a | Yes | 109 | | PSSRU 2018/2019 based on “Consultant: medical” | |
| Assistant/registrar radiologist | n/a | Yes | 47 | | PSSRU 2018/2019 based on “Registrar” | |
| Radiographer | n/a | Yes | 37.00 | | PSSRU 2018/2019 based on hospital based “scientific and professional staff”.  (Band 5) | |
| Surgical care practitioner | Yes | n/a | 65 | | PSSRU 2018/2019 based on “hospital based nurses”  (Band8a) | |
| Anaesthetic nurse | Yes | Yes | 47 | | PSSRU 2018/2019 based on “hospital based nurses”  (Band 6) | |
| Scrub nurse (table) | Yes | Yes | 38 | | PSSRU 2018/2019 based on “hospital based nurses”  (Band 5) | |
| Scrub nurse (floor) | Yes | Yes | 38 | | PSSRU 2018/2019 based on “hospital based nurses”  (Band 5) | |
| Healthcare assistant | n/a | Yes | 8.93 | | NHS Employers website 2018/2019 hourly rate  (Band 2 with 3-4 year experience) | |
| Perfusionist | Yes | n/a | 62.35 | | PSSRU 2018/2019 based on the average of Band 7 and Band 8a “hospital based scientific and professional staff” | |
| **Consumables Cost** | | | | | | |
| **Resource or unit intervention** | **OSR** | **TEVAR** | **Cost (£)** | | **Cost Source** | |
| Central lines | Yes | Yes | 25 | | Royal Papworth Hospital NHS Trust based on personal communication with Rosie Thornton between Nov 2019-20 | |
| Arterial lines | Yes | Yes | 25 | | Royal Papworth Hospital NHS Trust based on personal communication with Rosie Thornton between Nov 2019-20 | |
| Nasopharyngeal probe | Yes | n/a | 15 | | Royal Papworth Hospital NHS Trust based on personal communication with Rosie Thornton between Nov 2019-20 | |
| Urinary catheter | Yes | Yes | 36 | | Royal Papworth Hospital NHS Trust based on personal communication with Rosie Thornton between Nov 2019-20 | |
| Diathermy pad | Yes | n/a | 1.00 | | Royal Papworth Hospital NHS Trust based on personal communication with Rosie Thornton between Nov 2019-20 | |
| Swabs | Yes | Yes | 0.50 | | Imperial College Healthcare NHS Trust based on personal communication with Colin Bicknell between Jan-June 2020 | |
| Size 15 knife blade | Yes | n/a | 10 | | Royal Papworth Hospital NHS Trust based on personal communication with Rosie Thornton between Nov 2019-20 | |
| Size 10 knife blade | Yes | n/a | 10 | | Royal Papworth Hospital NHS Trust based on personal communication with Rosie Thornton between Nov 2019-20 | |
| Cell saver tubing and fluids | Yes | n/a | 235 | | Royal Papworth Hospital NHS Trust based on personal communication with Rosie Thornton between Nov 2019-20 | |
| Side towels | Yes | n/a | 1 | | Royal Papworth Hospital NHS Trust based on personal communication with Rosie Thornton between Nov 2019-20 | |
| Vicryl stay suture | Yes | n/a | 3.15 | | Royal Papworth Hospital NHS Trust based on personal communication with Rosie Thornton between Nov 2019-20 | |
| Cannula (all sorts) | Yes | n/a | 0.50 | | Royal Papworth Hospital NHS Trust based on personal communication with Rosie Thornton between Nov 2019-20 | |
| Axillary cannula | Yes | n/a | 0.50 | | Royal Papworth Hospital NHS Trust based on personal communication with Rosie Thornton between Nov 2019-20 | |
| 4/0 prolene | Yes | n/a | 7.30 | | Royal Papworth Hospital NHS Trust based on personal communication with Rosie Thornton between Nov 2019-20 | |
| 5/0 prolene | n/a | Yes | 7.30 | | Royal Papworth Hospital NHS Trust based on personal communication with Rosie Thornton between Nov 2019-20 | |
| 3/0 prolene | Yes | n/a | 3.15 | | Royal Papworth Hospital NHS Trust based on personal communication with Rosie Thornton between Nov 2019-20 | |
| Maxalon | Yes | n/a | 3.15 | | Royal Papworth Hospital NHS Trust based on personal communication with Rosie Thornton between Nov 2019-20 | |
| Monocryl suture | Yes | Yes | 25 | | Imperial College Healthcare NHS Trust based on personal communication with Colin Bicknell between Jan-June 2020 | |
| 2/0 vicryl | Yes | Yes | 3.15 | | Royal Papworth Hospital NHS Trust based on personal communication with Rosie Thornton between Nov 2019-20 | |
| By-pass circuit disposable bits | Yes | n/a | 650 | | Royal Papworth Hospital NHS Trust based on personal communication with Rosie Thornton between Nov 2019-20 | |
| Teflon pledgets/strips | Yes | n/a | 1 | | Royal Papworth Hospital NHS Trust based on personal communication with Rosie Thornton between Nov 2019-20 | |
| Bair hugger blanket | Yes | n/a | 6 | | Royal Papworth Hospital NHS Trust based on personal communication with Rosie Thornton between Nov 2019-20 | |
| Haemostatic adjuncts | Yes | n/a | 25 | | Royal Papworth Hospital NHS Trust based on personal communication with Rosie Thornton between Nov 2019-20 | |
| Sternal wires | Yes | n/a | 42 | | Royal Papworth Hospital NHS Trust based on personal communication with Rosie Thornton between Nov 2019-20 | |
| Biosyn | Yes | n/a | 3.15 | | Royal Papworth Hospital NHS Trust based on personal communication with Rosie Thornton between Nov 2019-20 | |
| Dressings | Yes | n/a | 0.50 | | Royal Papworth Hospital NHS Trust based on personal communication with Rosie Thornton between Nov 2019-20 | |
| Argyl drain | Yes | n/a | 5 | | Royal Papworth Hospital NHS Trust based on personal communication with Rosie Thornton between Nov 2019-20 | |
| Redivac drain | Yes | n/a | 5 | | Royal Papworth Hospital NHS Trust based on personal communication with Rosie Thornton between Nov 2019-20 | |
| Drain sutures braided nylon | Yes | n/a | 3.15 | | Royal Papworth Hospital NHS Trust based on personal communication with Rosie Thornton between Nov 2019-20 | |
| Spinal drain | n/a | Yes | 413 | | Imperial College Healthcare NHS Trust based on personal communication with Colin Bicknell between Jan-June 2020 | |
| Suction tube | n/a | Yes | 1 | | Imperial College Healthcare NHS Trust based on personal communication with Colin Bicknell between Jan-June 2020 | |
| Sheath | n/a | Yes | 10 | | Imperial College Healthcare NHS Trust based on personal communication with Colin Bicknell between Jan-June 2020 | |
| Angiography hollow needles | n/a | Yes | 2 | | Imperial College Healthcare NHS Trust based on personal communication with Colin Bicknell between Jan-June 2020 | |
| Pigtail catheter | n/a | Yes | 10 | | Imperial College Healthcare NHS Trust based on personal communication with Colin Bicknell between Jan-June 2020 | |
| J wire | n/a | Yes | 65 | | Imperial College Healthcare NHS Trust based on personal communication with Colin Bicknell between Jan-June 2020 | |
| Terumo wire | n/a | Yes | 5 | | Imperial College Healthcare NHS Trust based on personal communication with Colin Bicknell between Jan-June 2020 | |
| Super stiff Miere wire | n/a | Yes | 70 | | Imperial College Healthcare NHS Trust based on personal communication with Colin Bicknell between Jan-June 2020 | |
| Extension for injection pump | n/a | Yes | 9 | | Imperial College Healthcare NHS Trust based on personal communication with Colin Bicknell between Jan-June 2020 | |
| Iodinated contrast | n/a | Yes | 10 | | Imperial College Healthcare NHS Trust based on personal communication with Colin Bicknell between Jan-June 2020 | |
| Injection pump contract syringe | n/a | Yes | 15 | | Imperial College Healthcare NHS Trust based on personal communication with Colin Bicknell between Jan-June 2020 | |
| Measuring pigtail catheter | n/a | Yes | 12 | | Imperial College Healthcare NHS Trust based on personal communication with Colin Bicknell between Jan-June 2020 | |
| 12F Sheath | n/a | Yes | 7 | | Imperial College Healthcare NHS Trust based on personal communication with Colin Bicknell between Jan-June 2020 | |
| Saline | n/a | Yes | 5 | | Imperial College Healthcare NHS Trust based on personal communication with Colin Bicknell between Jan-June 2020 | |
| Sterile bowls for coiling wires | n/a | Yes | 30 | | Imperial College Healthcare NHS Trust based on personal communication with Colin Bicknell between Jan-June 2020 | |
| Moulding balloon | n/a | Yes | 300 | | Imperial College Healthcare NHS Trust based on personal communication with Colin Bicknell between Jan-June 2020 | |
| Syringe 20ml | n/a | Yes | 0.15 | | Imperial College Healthcare NHS Trust based on personal communication with Colin Bicknell between Jan-June 2020 | |
| Diathermy forceps | n/a | Yes | 195.80 | | Imperial College Healthcare NHS Trust based on personal communication with Colin Bicknell between Jan-June 2020 | |
| Blade | n/a | Yes | 5.89 | | Imperial College Healthcare NHS Trust based on personal communication with Colin Bicknell between Jan-June 2020 | |
| Surgicel | n/a | Yes | 55.82 | | Imperial College Healthcare NHS Trust based on personal communication with Colin Bicknell between Jan-June 2020 | |
| **Blood Products** | | | | | | |
| **Resource or unit intervention** | **OSR** | **TEVAR** | **Cost (£)** | | **Cost Source** | |
| Heparin | Yes | n/a | 14.85 | | BNF NICE 2018/2019 | |
| Protamine | Yes | n/a | 49.55 | | BNF NICE 2018/2019 | |
| Standard Red cells | Yes | Yes | 128.99 | | NHS Blood and Transplant Price list 2018/19 | |
| Platelets, Pooled | Yes | Yes | 185.86 | | NHS Blood and Transplant Price list 2018/19 | |
| Fresh Frozen Plasma | Yes | Yes | 28.46 | | NHS Blood and Transplant Price list 2018/19 | |
| Cryoprecipitate, Pooled | Yes | Yes | 177.55 | | NHS Blood and Transplant Price list 2018/19 | |
| Octaplex | Yes | n/a | 125 | | Royal Papworth Hospital NHS Trust based on personal communication with Priya Sastry in July 2020 | |
| Beriplex | Yes | n/a | 125 | | Royal Papworth Hospital NHS Trust based on personal communication with Priya Sastry in July 2020 | |
| Fibrinogen | Yes | n/a | 364 | | Royal Papworth Hospital NHS Trust based on personal communication with Priya Sastry in July 2020 | |
| Albumin | Yes | n/a | 42.50 | | Royal Papworth Hospital NHS Trust based on personal communication with Priya Sastry in July 2020 | |
| Novo7 | Yes | n/a | 919 | | Royal Papworth Hospital NHS Trust based on personal communication with Priya Sastry in July 2020 | |

**Table S5: Unit costs of resources and interventions post procedure up until discharge**

| **Resource or unit intervention** | **Cost (£)** | **Unit** | **Cost Source** |
| --- | --- | --- | --- |
| **Type of stay** | | | |
| ICU | 1417.63 | Per day | From<https://www.nice.org.uk/guidance/GID-NG10072/documents/evidence-review-12>inflated using PSSRU index Curtis L, Burns A. Unit Costs of Health and Social Care 2019. Canterbury: Personal Social Services Research Unit, University of Kent; 2019 |
| HDU | 724.18 | Per day | From <https://www.nice.org.uk/guidance/GID-NG10072/documents/evidence-review-12> inflated using PSSRU index Curtis L, Burns A. Unit Costs of Health and Social Care 2019. Canterbury: Personal Social Services Research Unit, University of Kent; 2019 |
| Ward | 416.90 | Per day | From <https://www.nice.org.uk/guidance/GID-NG10072/documents/evidence-review-12>, inflated using PSSRU index Curtis L, Burns A. Unit Costs of Health and Social Care 2019. Canterbury: Personal Social Services Research Unit, University of Kent; 2019 |
| **Blood products** | | | |
| Standard Red blood cells | 128.99 | Per unit | NHS Blood and Transplant Price list 2018/2019 |
| Platelets, Pooled | 185.86 | Per unit | NHS Blood and Transplant Price list 2018/2019 |
| FFP | 28.46 | Per unit | NHS Blood and Transplant Price list 2018/2019 |
| Cryoprecipitate, Pooled | 177.55 | Per unit | NHS Blood and Transplant Price list 2018/2019 |
| **Imaging** | | | |
| CT | 97 | Per scan | National Schedule of NHS costs 2018/19. Weighted Average of codes RD20A, RD21A, RD22Z to RD27Z |
| MRI | 341 | Per scan | National Schedule of NHS costs 2018/19. Weighted average of codes RD08Z to RD10Z |
| X-ray (plain films) | 31 | Per scan | National Schedule of NHS costs 2018/19. Code: DAPF |
| TOE | 257 | Per scan | National Schedule of NHS costs 2018/19. Code: EY50Z as “Complex Echocardiogram” |
| TTE | 257 | Per scan | National Schedule of NHS costs 2018/19. Code : EY50Z as “Complex Echocardiogram” |
| Echocardiogram | 64 | Per scan | National Schedule of NHS costs 2018/19. Code : RD51A as “Simple Echocardiogram, 19 years and over” |
| Ultrasound | 51 | Per scan | National Schedule of NHS costs 2018/19. Code : RD47Z “Vascular Ultrasound” |
| Renogram | 209 | Per scan | National Schedule of NHS costs 2018/19. Code : RN25A “Renogram, 19 years and over” |
| Fluoroscopy | 118 | Per scan | National Schedule of NHS costs 2018/19: average of RD30Z, RD31Z, RD32Z |
| Angiogram | 150 | Per scan | https://www.rcpjournals.org/content/clinmedicine/suppl/2020/05/18/20.3.e40.DC1/Balami_Supplementary_Material_S1.pdf |

**Table S6: Unit costs during follow-up**

| **Resource or unit intervention** | **Cost (£)** | **Units** | **Source** |
| --- | --- | --- | --- |
| **Primary / community care** | | | |
| GP visits (surgery) | 39 | Per average contact time 9.22 minutes | Unit Costs of Health and Social Care 2019 page 120 £39 per surgery consultation |
| GP visits (home) | 100.62 | Cost home visit (23.4 minutes including travel time) | *Unit Costs of Health and Social Care 2015 page 176 average home visit is 11.4 minutes with 12 minutes of travel time. Cost on 23.4 minutes of GP time  *Unit Costs of Health and Social Care 2019 page 119 £4.30 per minute |
| Nurse visit (surgery) | 42 | Per hour | *GP practice nurse, Unit Costs of Health and Social Care 2019 page 118 £42 per hour |
| Nurse visit (surgery) | 10.85 | Per contact time 15.5 minutes | *Unit Costs of Health and Social Care 2015 page 174 15.5 minute for contact. *Unit Costs of Health and Social Care 2019 page 118 £42 per hour |
| Nurse visit (home) | 16.38 | Per hour | *Unit Costs of Health and Social Care 2015 page 176 average home visit is 11.4 minutes with 12 minutes of travel time. Cost on 23.4 minutes of GP time. Assumed the travel and contact time is the same for a nurse as a GP.  *Unit Costs of Health and Social Care 2019 page 118 £42 per hour |
| Physiotherapy/Occupational therapy | 58 | Per unit | **NHS Reference costs 2018/2019, assumed Physiotherapy (outpatients code 650) |
| **Secondary care** | | | |
| A&E visits | 166 | Per visit | National Schedule of NHS costs 2018/19. Index “AE” |
| Outpatient Appointments Vascular Surgery (Consultant led) | 148 | Per appointment | National Schedule of NHS costs 2018/19. Service code : 107 in “total outpatient attendance” |
| Outpatient Appointments Cardiothoracic Surgery (Consultant led) | 241 | Per appointment | National Schedule of NHS costs 2018/19. Service code : 170 in “total outpatient attendance” |
| **Imaging** | | | |
| MRI | 341 | Per investigation | National Schedule of NHS costs 2018/19. Weighted average of codes RD08Z to RD10Z |
| CT | 97 | Per investigation | National Schedule of NHS costs 2018/19. Weighted Average of codes RD20A, RD21A, RD22Z to RD27Z |

**Table S7: Unit costs of hospital re-admissions**

| **Condition** | **HRG code** | **Cost (£)** |
| --- | --- | --- |
| Pleurisy | weighted average of DZ28Z and DZ28B (Pleurisy) | 365 |
| Chest pain | weighted averages of EB12A to EB12C (unspecified chest pain with CC score range 0-11+) | 400 |
| Cardiac event | weighted averages of EB10A to EB10E (actual or suspected myocardial infarction) | 1 478 |
| Infection and haematemesis | Weighted average WH07A to WH07b (Infections or other complications of procedures without and with single and with multiple interventions | 1 793 |
| Sepsis | weighted averages of WJ06A to WJ06J (sepsis without intervention, with intervention and with multiple intervention) | 2 206 |
| Elective angiography | weighted average of EY43A to EY43F (Standard cardiac cath) | 2 401 |
| Angiography | weighted averages of EY41A to EY41D (standard percutaneous transluminal coronary angioplasty) | 2 689 |
| Groin pseudoaneurysm | weighted averages of YR11A to YR11D (pertcut transluminal angioplasty of single blood vessel in CC score range) | 2 816 |
| Carotid -Subclavian bypass | weighted averages of YQ31A+YQ31B (single open procedure on carotid artery CC (0 - 5+) | 5 260 |
| Elective carotid-subclavian bypass | Elective weighted averages of YQ31A+YQ31B (single open procedure on carotid artery CC (0 - 5+) | 5 260 |
| Elective complex endovascular repair of abdominal aortic aneurysm | Elective weighted averages of YR66A to YR67B (standard endovascular repair of AAA and complex) | 7 321 |
| Endovascular repair of abdominal aortic aneurysm | weighted averages of YR66A to YR67B (standard endovascular repair of AAA and complex) | 7 499 |
| Elective Open surgery repair of abdominal aortic aneurysm | Elective weighted average of open repair of AAA single and multiple open procedures | 9 141 |
| Endovascular repair of, Thoracic or Thoracoabdomminal AA (fenestrated) | weighted averages of YR62A+YR62B+YR63A+YR63B | 9 314 |
| Complex repair of descending thoracic aorta (fenestrated) | weighted averages of YR62A+YR62B+YR63A+YR63B | 9 314 |
| Endovascular repair of, Thoracic or Thoracoabdominal AA | weighted average of YR61Z and YR60Z (standard and complex endovascular repair of Thoracoabdominal Aortic Aneurysm using Branched Stent Graft | 11 856 |
| Elective Complex endovascular repair of Thoracic or Thoracoabdominal aortic aneurysm | Elective weighted average of YR61Z and YR60Z (standard and complex endovascular repair of Thoracoabdominal Aortic Aneurysm using Branched Stent Graft | 12 493 |

**S8: Table of participants from ETTAA study sites**

| **Centre** | **TEVAR (n=115)** | | | **OSR (n=35)** | |
| --- | --- | --- | --- | --- | --- |
|  | Aortic arch endovascular repair (n=2) | Complex repair thoracoabdominal stent with fenestrated or branch grafting (n=16) | Descending thoracic aortic repair (n=97) | OSR (less costly surgical grafts) (n=22) | OSR (more costly surgical grafts) (n=13) |
| Addenbrookes | 0 | 1 | 5 | 0 | 1 |
| Bedford | 0 | 0 | 1 | 0 | 1 |
| Birmingham | 0 | 0 | 1 | 0 | 0 |
| Blackpool | 0 | 0 | 1 | 4 | 0 |
| Brighton & Sussex | 0 | 0 | 7 | 0 | 0 |
| Bristol | 0 | 0 | 9 | 1 | 1 |
| Central Manchester |  | 2 | 2 | 0 | 0 |
| Derby | 0 | 0 | 0 | 0 | 0 |
| Glenfield | 0 | 0 | 3 | 0 | 0 |
| Guys & St Thomas | 1 | 1 | 5 | 2 | 0 |
| Hull | 0 | 0 | 0 | 0 | 0 |
| Imperial | 1 | 4 | 13 | 4 | 0 |
| Kings | 0 |  | 0 | 0 | 0 |
| Leeds | 0 | 2 | 7 | 2 | 1 |
| Liverpool Heart & Chest | 0 | 0 | 0 | 3 | 0 |
| Musgrove Park | 0 | 0 | 1 | 0 | 0 |
| Newcastle | 0 | 0 | 6 | 0 | 0 |
| Norfolk & Norwich | 0 | 0 | 5 | 1 | 0 |
| North Cumbria | 0 | 0 | 1 | 0 | 0 |
| Papworth | 0 | 0 | 2 | 1 | 2 |
| Plymouth | 0 | 0 | 0 | 0 | 2 |
| Royal Free | 0 | 0 | 5 | 0 | 0 |
| Royal Liverpool | 0 | 2 | 2 | 0 | 0 |
| Sheffield | 0 | 0 | 0 | 0 | 1 |
| South Manchester | 0 | 0 | 2 | 0 | 0 |
| South Tees | 0 | 0 | 2 | 0 | 0 |
| Southampton | 0 | 1 | 5 | 3 | 4 |
| St George’s | 0 | 3 | 11 | 0 | 0 |
| Surrey & Sussex | 0 | 0 | 0 | 0 | 0 |
| York | 0 | 0 | 1 | 1 | 0 |
